# Correction to “Salinity Trends in a Groundwater System Supplemented by 50 Years of Imported Colorado River Water”

**DOI:** 10.1021/acsestwater.3c00665

**Published:** 2023-11-07

**Authors:** Jennifer S. Harkness, Patrick M. McCarthy, Bryant C. Jurgens, Zeno F. Levy

Perchlorate concentrations were incorrectly reported as milligrams per liter in the original manuscript. The units need to be corrected to micrograms per liter (μg/L) in lines 275 and 279 and Figure 3. The units also need to be corrected in Figure S5 of the Supporting Information. A corrected version of Figure 3 and the corrected Supporting Information are provided.

**Figure 3:**
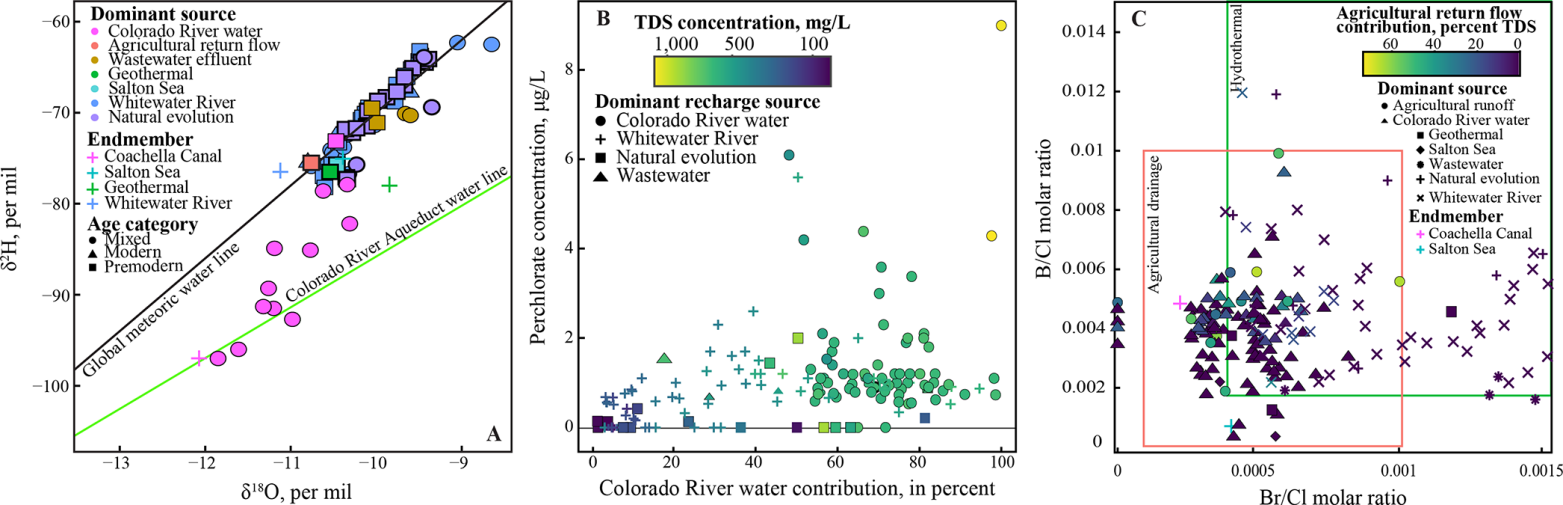
Geochemical and isotopic relations supporting the modeled geochemical results. (A) Water isotopes of oxygen (δ^18^O) and hydrogen (δ^2^H) in wells, with color indicating the modeled dominant water source and shape indicating the groundwater age in the well. Wells with predominantly Colorado River water and agricultural return flow plot between the global meteoric water line (GMWL) and the evaporative Colorado River Aqueduct water line.^8^ (B) Relation between increasing perchlorate concentrations with increasing modeled Colorado River water contributions and TDS concentrations due to a known perchlorate contamination source in the lower Colorado River.^81,82^ (C) Boron/chloride (B/Cl) and bromide/chloride (Br/Cl) molar ratios in wells with variations in modeled contributions from agricultural return flow, showing lower ratios for wells with predominantly agricultural return and Colorado River water.^86^

The last paragraph that begins on page 3257 should read as follows: CRW contributions were significantly correlated with perchlorate concentrations (ρ = 0.48, *p* < 0.0001; Figure 3). Perchlorate contamination of the lower Colorado River was identified in 1997, and concentrations have since steadily decreased; the concentration in the Coachella Canal endmember was 0.9 μg/L.^64,81–83^ Lake Cahuilla (Figure 1) receives CRW from the Coachella Canal and is subject to severe evaporation, reaching rates of 1800 mm/year (71 in./year).^84^ Wells with perchlorate concentrations greater than 2 μg/L were predominantly CRW and located adjacent to Lake Cahuilla and the Whitewater and Thomas E. Levy GRFs (Figure 3 and Figure S4). Perchlorate was correlated to tritium concentrations (ρ = 0.81, *p* < 0.0001), supporting a recent (<50 years), external source of perchlorate to groundwater. In wells with perchlorate measurements sampled by the USGS (*n* = 161; 55), the proportions of wells with concentrations above 0.5 and 1 μg/L were 22% and 11%, respectively, which exceeded the expected proportions of perchlorate from atmospheric deposition (14% and 4.7%, respectively) in the arid Indio subbasin.^85^ Some public-supply wells had results for perchlorate in the SWRCB Division of Drinking Water database;^52^ however, the reporting limits for these data were either 2 or 4 μg/L, so these results were not included in the calculation to eliminate any bias from wells reported as nondetections.

## Supporting Information Available

Additional description of methods with supporting tables and figures, including TDS concentrations in end member samples; summary statistics of relations between TDS and geochemical tracers; relation between measured TDS concentrations and specific conductance in samples; location of wells modeled for TDS source contributions; maps of well densities and areal proportions of TDS concentration categories; perchlorate concentration in sample wells and its relation to CRW contributions; locations of wells with dominant contributions of treated wastewater, agricultural return flow, Salton Sea water, and geothermal brines; and time series plots of areal proportions of TDS concentrations in the shallow and deep aquifers (PDF)

