# Supplementary material for: Correction to “Salinity Trends in a Groundwater System Supplemented by 50 Years of Imported Colorado River Water”
Source: ACS ES T Water. 2023 Nov 7;3(12):4195–6. doi: 10.1021/acsestwater.3c00665 (PMC10714391; doi:10.1021/acsestwater.3c00665)
Supplement: Supplementary file 1 — ew3c00665_si_001.pdf [file ew3c00665_si_001.pdf]

Salinity trends in a groundwater system supplemented by 50 years of imported Colorado River Water.

Jennifer S. Harkness[a*1], Patrick M. McCarthy[a,b], Bryant C. Jurgens[a], Zeno Levy[a]
[a]U.S. Geological Survey, California Water Science Center, Sacramento, CA USA
[b]Geology Department, California State University, Sacramento, Sacramento CA USA

Supporting Information

1. Additional Methods Descriptions

1.1 Conductance to TDS conversion

A conversion factor of 0.656 was derived from linear regression of samples with measured specific conductance and TDS values (Figure S1). Specific conductance values ranged from 29 to 23,000 µS/cm (at 25°C), and TDS concentrations ranged from 55 to 16,500 mg/L. (1) The derived conversion factor is consistent with previously reported values for natural waters. (2)

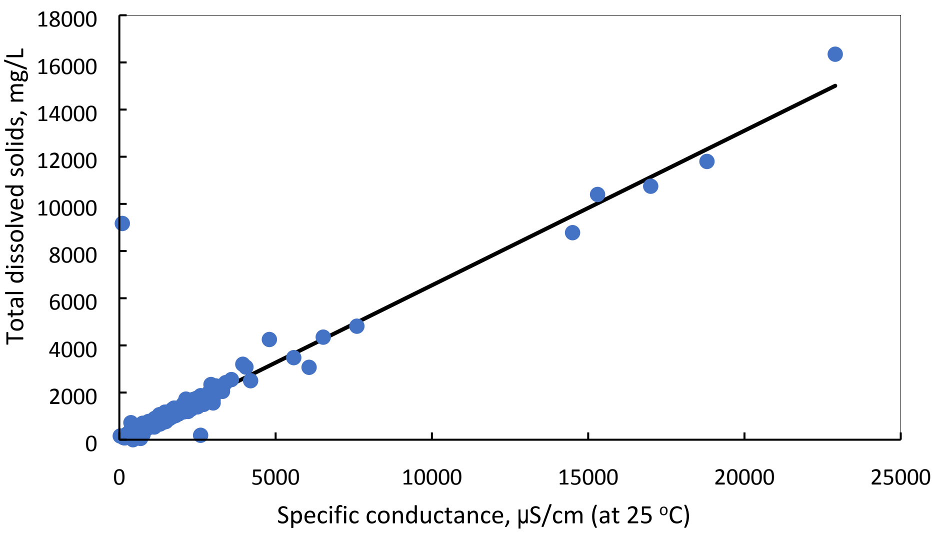

Figure S1. Linear regression results for relation between TDS and conductance in groundwater samples from the Indio subbasin, Coachella Valley, California with both measurements available. (1)

1.2 Inverse Geochemical Modeling

The study modeled the volumetric contribution of any of seven potential mixing endmembers on selected samples within the study using PHREEQC's inverse modeling. (3) The inverse modeling determined sets of mole transfers of phases and groundwater mixing sources that account for changes in water chemistry between one or a mixture of initial waters and a final solution. The model produced a series of linear equation that predicts the volumetric contribution

from each of seven endmembers that produce the chemical composition of the sample. The primary phases considered in the inverse modeling were water ($H2O_{(g)}$), amorphous silica (SiO_2) and ion-exchange reactions involving Ca, Na, K, and Mg. In the case where these conditions did not produce a model solution, additional mineral phases were added to the model to account for different flow paths and geochemical processes within the study area. While inverse modeling attempts to find the optimum mixing and mole transfer solution for each sample, it often output many possible model solutions for each sample. TDS contribution from each endmember were calculated from the modeled volumetric contribution, endmember TDS concentration, and addition or removal of water allowed as evaporation or dilution. Herein we reported the median volumetric and TDS contribution of each endmember. The full dataset and metadata are available in McCarthy et al. (4)

1.2.1 Dataset descriptions

The inverse geochemical model was developed and calibrate using 68 samples from 64 wells collected by the U.S. Geological Survey (USGS) for this study (5) and the California State Water Resources Control Board's (SWRCB) Groundwater Ambient Monitoring and Assessment Priority Basin Project (GAMA-PBP) (6), which included 11 public-supply and community wells, 21 irrigation or commercial wells (including wells screened to depths used by public-supply wells), 25 domestic and state small system wells, and 7 monitoring wells distributed throughout the Indio subbasin (Figure S2*A*). We modeled the volumetric mixing and geochemical evolution in two subsets of the full compiled dataset in Harkness (1). The first set is comprised of the most recent measurement for any well sampled after 2010, and included major ion chemistry (Ca, Mg, Cl^-, Na, K, Alkalinity, $SO4^-$, Si, and NO_3^{2-}) for 188 samples (Figure S2*B*) collected between 2010 and 2021 from 121 public-supply and community wells, 8 irrigation or commercial wells, 27 domestic wells and 31 monitoring wells (1) by the U.S. Geological Survey (USGS; 7), the California State Water Resources Control Board Division of Drinking Water (8), the California Department of Water Resources (DWR; 9), and the Coachella Valley Water District (10) (Figure S2). The second set is comprised of wells with continuous decadal sampling intervals of 1980 through 2000 or 1990 through 2010 and included major ion chemistry for 1382 samples across 131 wells (Figure S2*C*), collected between 1962 and 2021 from 121 public-supply wells and 10 monitoring wells by the USGS (7) and the California State Water Resources Control Board

Division of Drinking Water (8) (Figure S2). Both datasets were restricted to wells within the Indio subbasin (Figure S2).

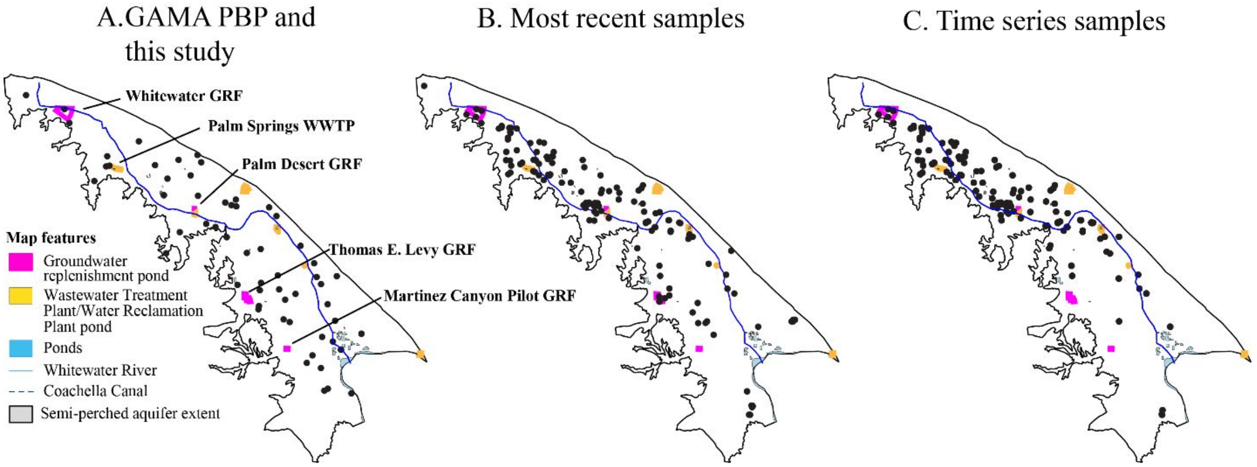

Figure S2. Locations of wells modeled for salinity source contributions in the Indio subbasin, Coachella Valley, California. (1) Locations of ponds at groundwater replenishment facilities (GRF), wastewater treatment and water reclamation plants (WWTP), and surface-water sources are shown on the maps.

In both datasets, silica was the limiting chemical constituent and was unreported in 130 and 1862 samples for the first and second datasets, respectively. (1) For these samples, modeled silica concentrations were used in place of measured concentrations. To model the silica concentrations, a PHREEQC model calculated the equilibrium concentration of silica in each sample using the silica saturation index (SSI). To compute the silica saturation index, an initial PHREEQC model was run on a dataset consisting of 79 GAMA-PBP samples from the study area with complete major ion chemistry data, including measured silica. The goal of this model was to calculate the SSI for samples with measured silica values and develop a linear regression model of the relation between SSI and pH. Using this regression model, the SSI for samples with no silica data could then be calculated using the measured pH for each sample and input into the PHREEQC equilibrium model to determine modeled silica concentrations. This approach allowed us to define the relation between pH and SSI with the function:

$$Y = -0.17(pH) + 0.58; R^2 = 0.60 \quad (1)$$

To verify the accuracy of this modeling approach, the modeled total dissolved solids (TDS) in mg/L was compared against the measured TDS for each sample. This relation is defined by the linear regression equation:

$$Y = 0.96x + 82.09; R^2 = 0.9995 \quad (2)$$

Then, the TDS for each sample was recalculated using the modeled silica concentrations in place of the measured, and the regression analysis repeated. The relation between the modeled TDS and measured TDS following this approach is defined by the linear regression equation:

$$Y = 0.96x + 84.18, R^2 = 0.9994 \quad (3)$$

1.2.2 Endmember Selection

Sources of mixing within the groundwater system considered were freshwater recharge, naturally evolved groundwater, imported Colorado River water, Salton Sea water, geothermal water, wastewater treatment plant effluent, and agricultural return flow. The samples representative of the natural groundwater evolution, freshwater recharge, imported Colorado River water, and the Salton Sea were selected from samples analyzed and reported by the USGS GAMA-PBP project. (5,6) The surface water sample, Indio-SW-1, was collected from the Whitewater River north of the study area. The Whitewater River is fed from regional precipitation and is the major drainage course in the Indio subbasin. Imported Colorado River water was sampled from the Coachella Canal (Indio-SW-2). This sample is geochemically similar to water sampled from Colorado River Aqueduct but includes a more extensive set of analyzed constituents. Sample Indio-GW-14 was determined to represent the natural geochemical evolution of groundwater through water-rock interactions due to the relatively high concentrations of sodium, potassium, and alkalinity. (5) S11-CODA-03U was a well sampled as part of the GAMA-PBP domestic well study in Coachella Valley that was known to contain Salton Sea water. (6) This sample was used in place of data available for the main Salton Sea because it included are more extensive set of geochemical constituents for the model. Robison (11) reported water chemistry results for 10 geothermal wells from the Oasis geothermal area. Of these wells, Well 9 was selected as the representative endmember because the reported TDS concentration was the median value of the dataset. (11) Geothermal endmembers within the study area were otherwise sparse due to a lack of published, recently analyzed samples. Groundwater chemistry from a shallow monitoring well located near the Palm Springs wastewater treatment plant (PS-MW1) was used to represent the wastewater effluent chemistry because it matched publicly available (but incomplete) data reported for wastewater effluent

within the study area. (1) Groundwater chemistry from a shallow monitoring well screened within the semi-perched aquifer (TEL-GRF-MW-9) was used to represent the agricultural return flow as the semi-perched aquifer water is derived from agricultural drainage. (1)

Table S1. TDS concentrations (mg/L) for each of the inverse model endmembers measured as part of this study or reported in previous studies.

**Endmember**	**Reported TDS, mg/L**	**Source**
Natural Groundwater Evolution	224	Soldavini et al. (6)
Coachella Canal	675	Soldavini et al. (5)
Salton Sea	8780	Soldavini et al. (5)
Whitewater River	198	Soldavini et al. (6)
Geothermal	1470	Robison (10)
Wastewater Effluent	800	Harkness (1)
Agricultural Runoff	2100	Harkness (1)

1.2.3 Model Design

The primary phases considered in the inverse modeling were water ($H_2O_{(g)}$), amorphous silica (SiO_2), fertilizer as calcium nitrate ($Ca(NO_3)_2$), $CO_{2(g)}$, calcite, plagioclase, organic matter as CH_2O, and ion-exchange reactions involving Ca, Na, K, and Mg. All phases were permitted to dissolve into or precipitate out of solution to simulate naturally occurring geochemical processes. In particular, the addition of $CO2_{(g)}$ and calcite was necessary to allow the surface water from the Colorado River endmember to interact with carbonate species in the unsaturated zone. The inclusion of organic matter accounted for microbial processes in the unsaturated zone that may be removing oxygen from the system. For samples in which these conditions did not produce a model solution, halite was added to the phases to account for evaporation and increased mixing from the more saline endmembers.

The uncertainty for all endmembers, elements, and geochemical phases was set at 10%, and the tolerance for the optimizing solver was set to $1x10^{-10}$. The model was constrained to reduce the output models to the minimum number of phases that could satisfy all the above constraints within the specified uncertainty limits. The PHREEQC "-minimal" modeling keyword minimizes the number of calculations that would be performed and produces the models that contain the only most essential geochemical reactions. (3)

During model calibration, we found that differences in analytical precision between reported constituents could result in computational rounding errors, preventing samples from being successfully modeled. To account for this, all chemical constituents were first rounded to equal degrees of precision prior to executing the model. Reported results were limited to two significant figures.

The mixing fractions determined by the inverse model are used to represent the relative volumetric contribution of each endmember to the final solution geochemistry. This mixing fraction, however, is not representative of the total dissolved solids (TDS) contribution of each endmember to the final solution TDS concentration. To calculate the TDS contribution of each endmember, the volumetric mixing fraction of each endmember is multiplied by the measured TDS concentration of the respective endmember (Table S1):

$$TDS_C = TDS_E * MixingFraction_E \quad (4)$$

where TDS_c is the contributed TDS in mg/L, TDS_E is the reported TDS for each endmember in mg/L, and $MixingFraction_E$ is the modeled volumetric mixing fraction of a given endmember. The TDS contributions of each endmember are then summed to give the modeled total TDS of the final solution. The percent difference between the total contributed TDS and the reported solution TDS was calculated and is used as a metric to evaluate each model's viability.

To evaluate the contribution of evaporation or dilution in each model, the sums of the mixing fractions with and without $H_2O_{(g)}$ mole transfer were calculated. Furthermore, the mass of water added to or removed from the models is calculated by multiplying the molar transfer of $H_2O_{(g)}$ by the molar mass, 0.01802 kg/mol. Evaluation of the mass transfer of water is a necessary check to account for mixing fractions greater than 1, which signal evaporation is also occurring within the model, or models where the mixing fractions do not sum to 1, indicating that water has been derived by mineral reactions in the mole-balance model.

1.2.4 Removal of non-viable models

Prior to post-processing, models that did not accurately represent the geochemistry of the solution were removed from the dataset. This filtering first accounted for models with model errors outside of an acceptable range for the study. This included individual models where the sum of the residuals was greater than 30 or the maximum fractional error was greater than 10%. Additionally, all models that involved sodium mole transfer (NaX) greater than -0.001 were

removed from the dataset. During calibration, we found that each time a saline solution was used in the model, the model would adjust for the abundance of sodium by precipitating it on the NaX parameter. In these scenarios, the sodium exchange values were outside of realistic amounts, and the models would not produce viable solutions. The filtering schema also removed individual models with a TDS percent difference greater than 25% and models resulting in total TDS contributions less than 0. As a final step, models that included greater than 30 moles H_2O (dilution) and less than -30 moles H_2O (evaporation) were removed. This is because the conceptual model requires that recharge occur primarily through mixing with the freshwater recharge endmember. While evaporation plays an important role in the system, putting a constrain on the model restricts the evaporation to realistic values.

In some instances, it was found that the above filtering schema was too aggressive and removed viable models from the dataset. Additionally, these models were often critical components in the time series dataset and would result in an incomplete time series analysis if removed. Therefore, a less aggressive filtering schema was applied to these samples when necessary. This filtering schema was the same as outlined above, but the steps to remove models with TDS percent difference greater than 25% and the steps to remove samples with H_2O mole transfers exceeding the acceptable limits were omitted. These samples are indicated in the "Method" column of the associated data release. (4)

1.2.5 Model Aggregation

Inverse modeling attempts to find the optimum mixing and mole transfer solution for each sample but often outputs many possible model solutions for each sample. Typically, one inverse model is chosen for each sample to best represent the set of geochemical reactions that explain the chemistry of the groundwater. However, given the size and complexity of the dataset, potential solutions could not be evaluated for every sample. To summarize the set of models after filtering for each sample, we took the median of the model output parameters as the representative result for each sample. During this aggregation step, we also determined the "Dominant Endmember," which is the mixing endmember with the highest median volumetric mixing fraction, and the "Dominant Contributor," which is the endmember that contributed the highest median TDS to each sample. In some scenarios, a relatively low TDS endmember may contribute a greater volume to the final solution, but a higher TDS endmember contributes a

greater mass of TDS, as represented by disparities between these two fields. As a final filtering step, aggregated samples where the highest median volumetric contribution was less than 20% were removed from the dataset. These samples involved too little contribution from the mixing endmembers and too great a contribution from mineral phases to appropriately address the study goals and were therefore deemed nonviable. Model aggregation also included the determination of the percent contribution of the Colorado River water to the total TDS of the sample. This value is calculated for each sample as the median volumetric contribution of the Colorado River water endmember; however, the calculation only considers models with a volumetric mixing fraction for Colorado River water greater than 0. The final results and metadata are reported in McCarthy et al. (4).

1.3 Data Analysis

Table S2. Spearman's rho coefficients for significant ($p<0.5$) correlations between total dissolved solids (TDS), geochemical model recharge contributions of Colorado River water (%CRW), agricultural return flow (%AgRF), treated wastewater (%WWTP), Salton Sea water (%SS) and geothermal brine (%Geo) and geochemical (nitrate [NO_3], perchlorate, [ClO_4],boron/chloride and bromide/chloride molar ratios), isotope ratios of oxygen ($\delta^{18}O$) and hydrogen ($\delta^{2}H$) and groundwater age tracers tritium (TU) and carbon-14 (C-14).

	NO_3	ClO_4	$\delta^{18}O$	$\delta^{2}H$	B/Cl	Br/Cl	%CRW	%AgRF	%WWTP	%SS	%Geo	TU	C-14
TDS	0.08	0.16					0.71	0.43	0.13	0.84	0.18		-0.29
NO_3		0.16			-0.06	0.16	-0.1	0.16	0.29				0.39
ClO_4			-0.34	-0.48	-0.1	-0.34	0.48		0.23		0.27	0.81	
$\delta^{18}O$				0.95		0.40	-0.42					-0.52	
$\delta^{2}H$						0.50	-0.57					-0.67	
B/Cl						0.27	-0.09		-0.1		0.90		-0.37
Br/Cl							-0.53					-0.31	0.32
%CRW								0.17	0.16	0.06	0.15	0.56	
%AgRF										0.10	0.06		-0.29
%WWTP											0.06		0.32
%SS													-0.26
%Geo													
TU													0.30
C-14													

1.4 Transect mapping and Two-dimensional interpolation

Hydrogeochemical transects were developed from regional TDS and well-construction data (Figure S7). (1) Transects were initially defined by a start and endpoint using coordinates projected using Albers Equal Area Conic Projection in meters with respect to the North

American Datum of 1983. All wells with TDS measurements and construction information (both depth to top and bottom of screened or open interval) within a 1.5 km lateral buffer distance from defined transect lines were used to construct hydrogeochemical transects. For wells with more than one TDS measurement within a given period of interest, the most recent measurement was used.

Well locations within a transect buffer area were "snapped" to the closest perpendicular point on the transect line, and distances from the transect start point to laterally projected well locations were calculated in R[1] using the sf package. (12) Land-surface elevation at all transect well locations were extracted from the U.S. Geological Survey 30-m resolution National Elevation Dataset (13) and are reported in meters above the North American Vertical Datum of 1988 (NAVD 88). Depths to the top, bottom, and midpoint of screened or open intervals were calculated by subtracting construction depths (reported relative to the land surface datum at the wellhead) to land-surface elevations at those points.

Two-dimensional interpolations of TDS measurements with respect to well depth and lateral distance from the transect start point with multilevel B-splines in R using the MBA package. (14) Well depths are represented here by the elevation of the midpoint of the screened or open interval with respect to NAVD 88. Interpolated TDS values were projected onto a 200 m by 200 grid m bounded by the most extreme well location values in vertical and lateral directions. Only interpolated values within the convex hull bounded by measured data in the vertical and lateral directions are shown. Two-dimensional interpolated TDS cross-sections were converted back into the projected coordinate system for three-dimensional representation in R using the plotly package. (15)

2. Supporting Figures

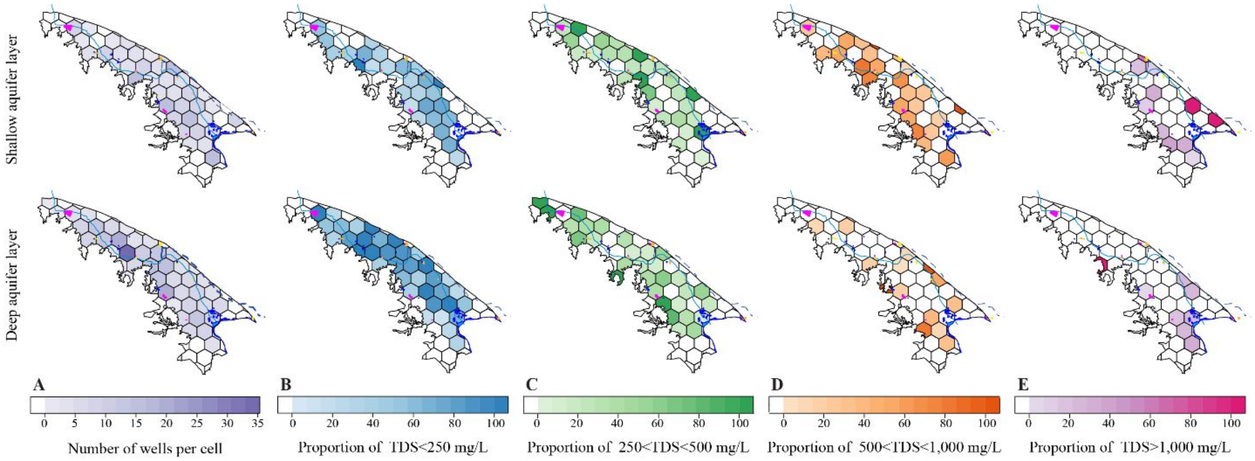

Figure S3. Well density maps of TDS concentration categories in 25 km^2 hexagonal grids of the shallow and deep aquifer layers of the Indio subbasin, Coachella Valley, California. Locations of groundwater replenishment ponds (pink), wastewater treatment and wastewater reclamation ponds (orange), and surface water sources (blue), are shown on the maps.

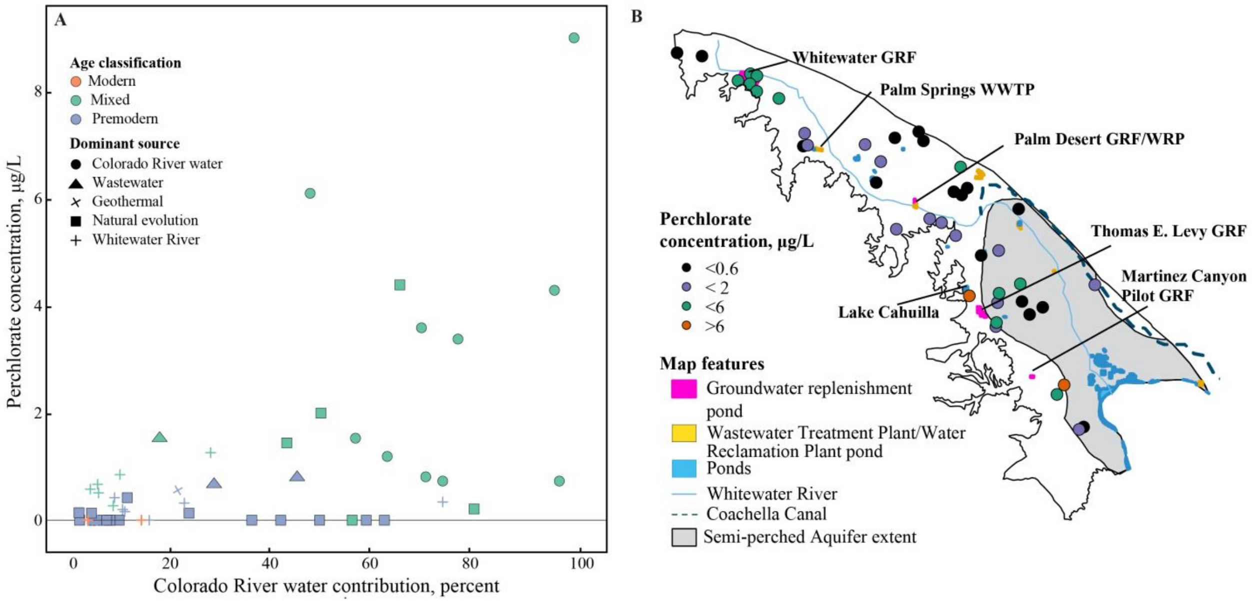

Figure S4. Perchlorate concentrations in wells in the Indio subbasin, Coachella Valley, California. (A) Relation between perchlorate and Colorado River water contributions by age classification. Waters with mixed aged water and higher contributions from of Colorado River water (16) have higher perchlorate concentration. (B) Map of wells with perchlorate detections.

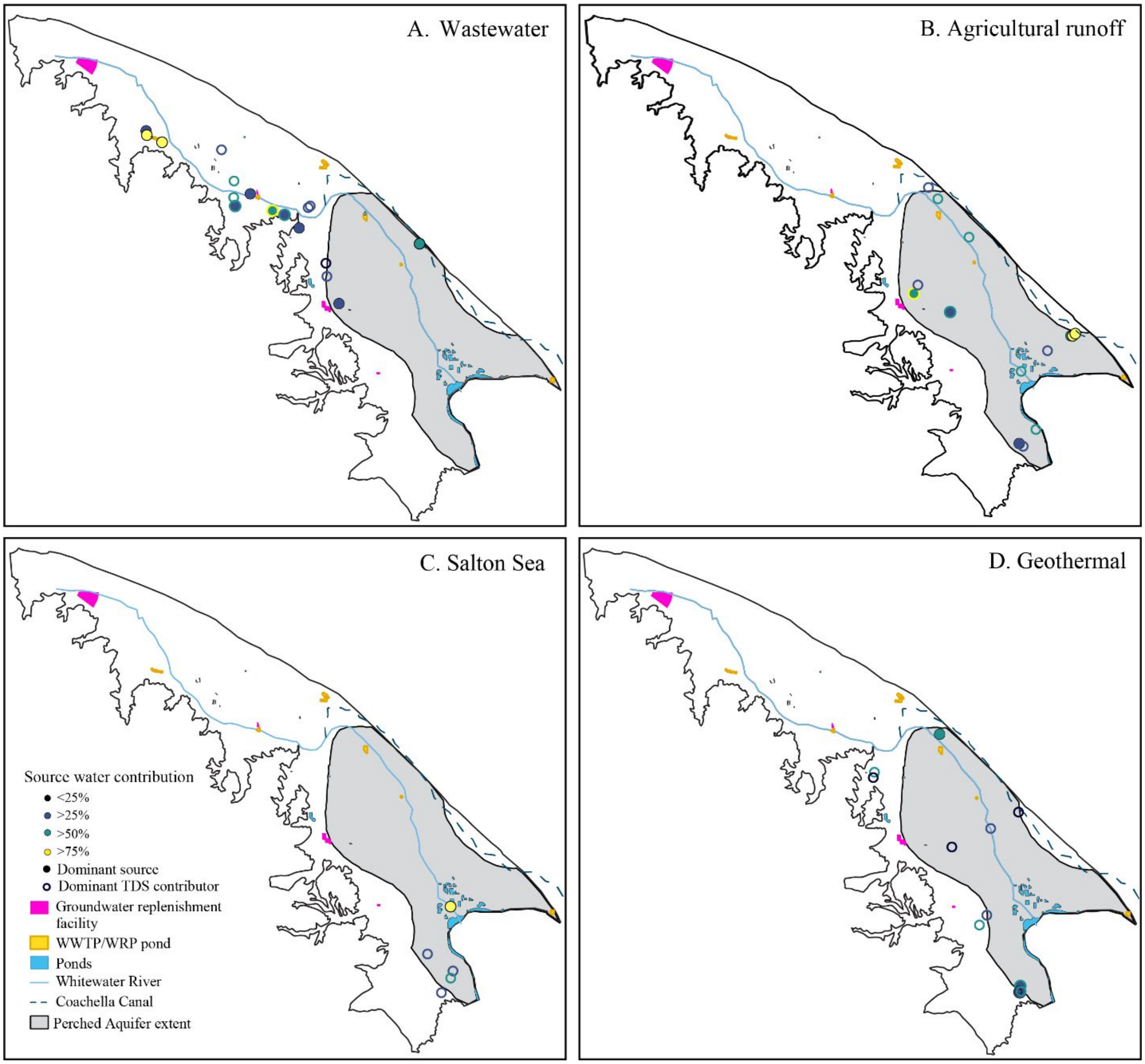

Figure S5. Maps of locations and modeled contribution proportion of (A) wastewater effluent (B) agricultural return flow, (C) Salton Sea water, and (D) geothermal brines in the Indio subbasin, Coachella Valley, California.

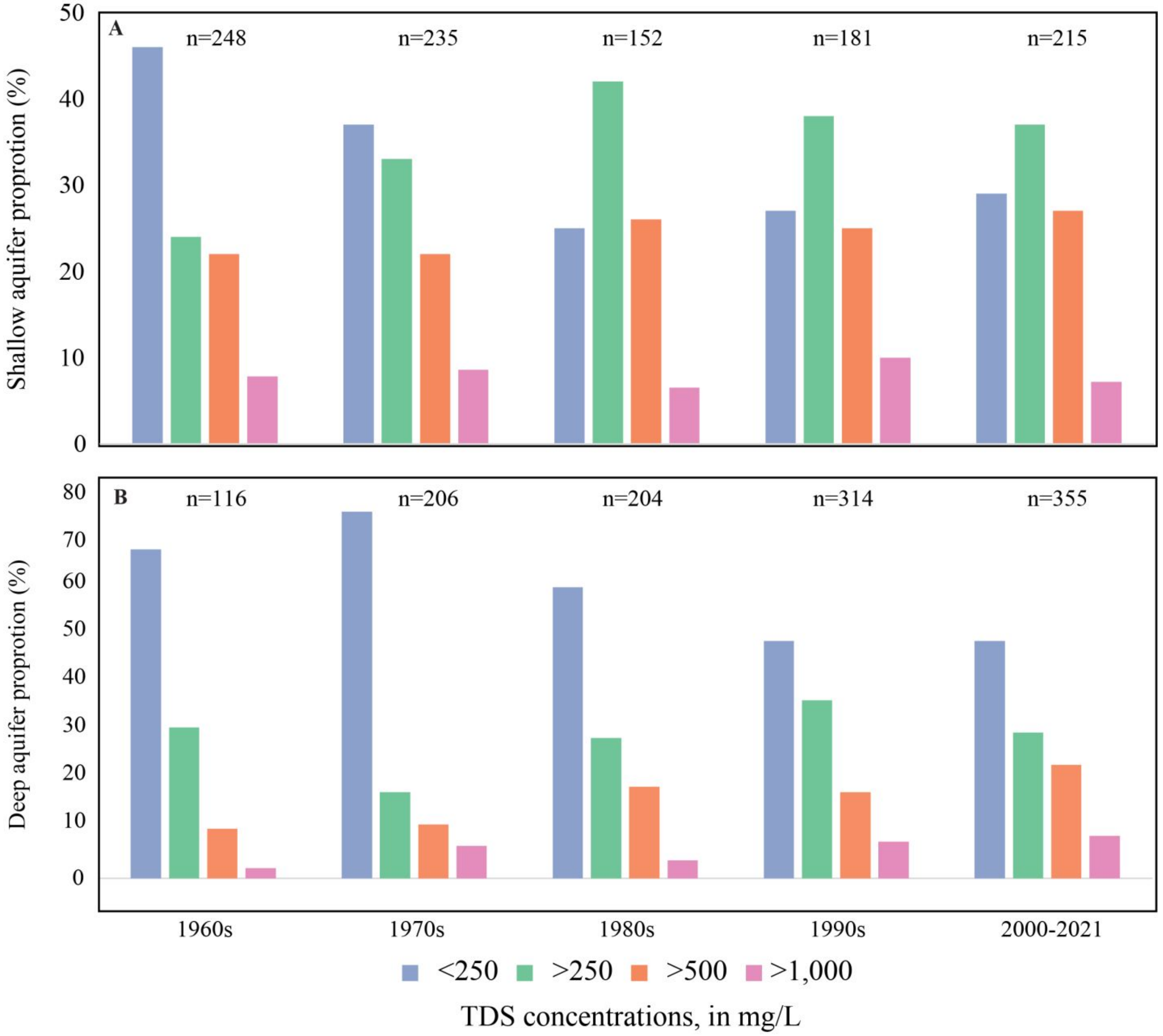

Figure S6. Aquifer proportion of TDS concentration categories through time in the (A) shallow and (B) deep aquifer layer of the Indio subbasin, Coachella Valley, California. To maintain similar areal coverage in each decade, different populations of wells were used in the areal proportion calculations, and wells sampled in the 2000s and 2010s were combined due to overall fewer wells sampled for TDS after 2000. However, the proportion of well types (domestic, public supply, irrigation, and production) and the distribution of well depths did not vary significantly through the decades. Monitoring wells were not included in the areal proportion calculations because the proportion of shallow monitoring wells near known high TDS sources increased through time

.

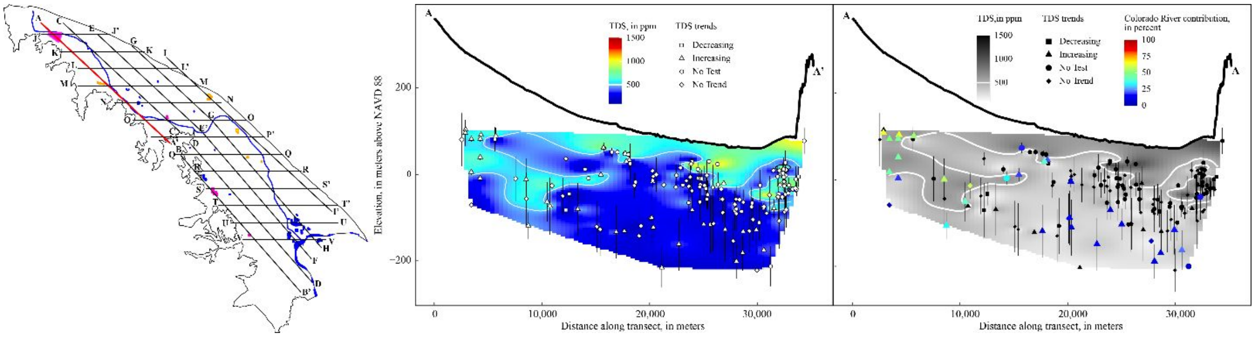
A
A'
TDS, in ppm
1500
1000
500
TDS trends
Decreasing
Increasing
No Test
No Trend
Colorado River contribution, in percent
100
75
50
25
0
Elevation, in meters above NAVD 88
200
0
−200
Distance along transect, in meters
0
10,000
20,000
30,000

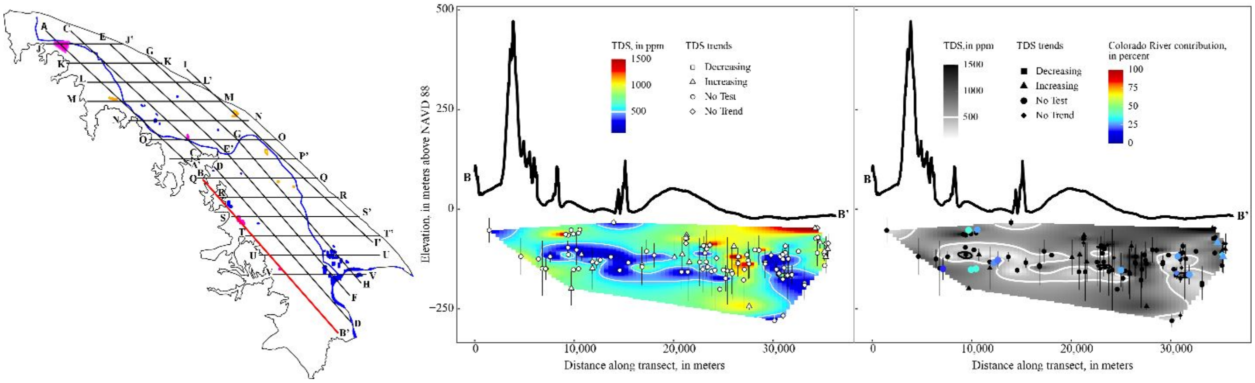
B
B'
TDS, in ppm
1500
1000
500
TDS trends
Decreasing
Increasing
No Test
No Trend
Colorado River contribution, in percent
100
75
50
25
0
Elevation, in meters above NAVD 88
500
250
0
−250
Distance along transect, in meters
0
10,000
20,000
30,000

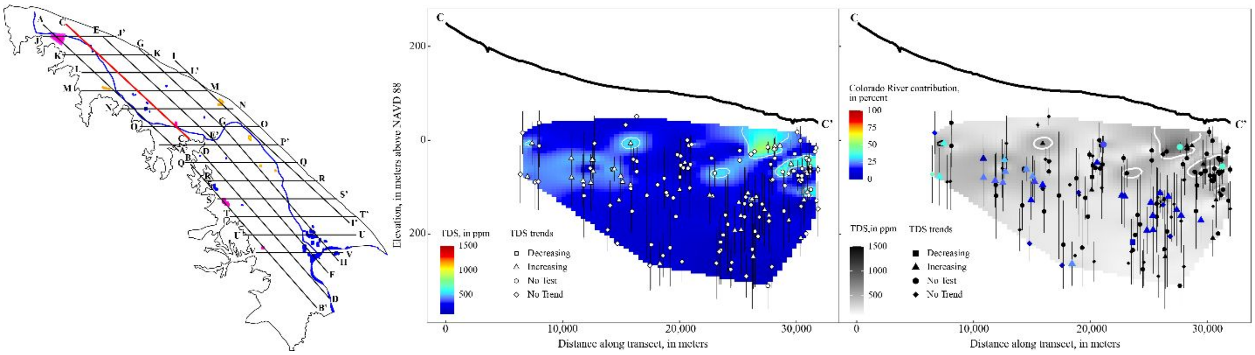
C
C'
Colorado River contribution, in percent
100
75
50
25
0
TDS, in ppm
1500
1000
500
TDS trends
Decreasing
Increasing
No Test
No Trend
Elevation, in meters above NAVD 88
200
0
200
Distance along transect, in meters
0
10,000
20,000
30,000

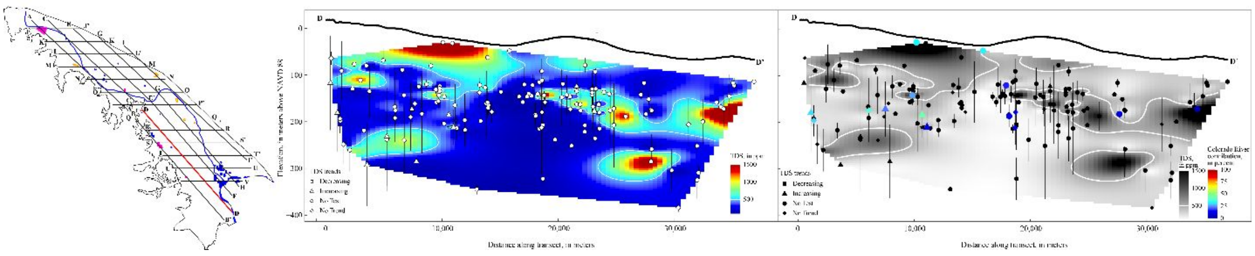
D
D'
Elevation, in meters above NAVD 88
Distance along transect, in meters
0
10,000
20,000
30,000

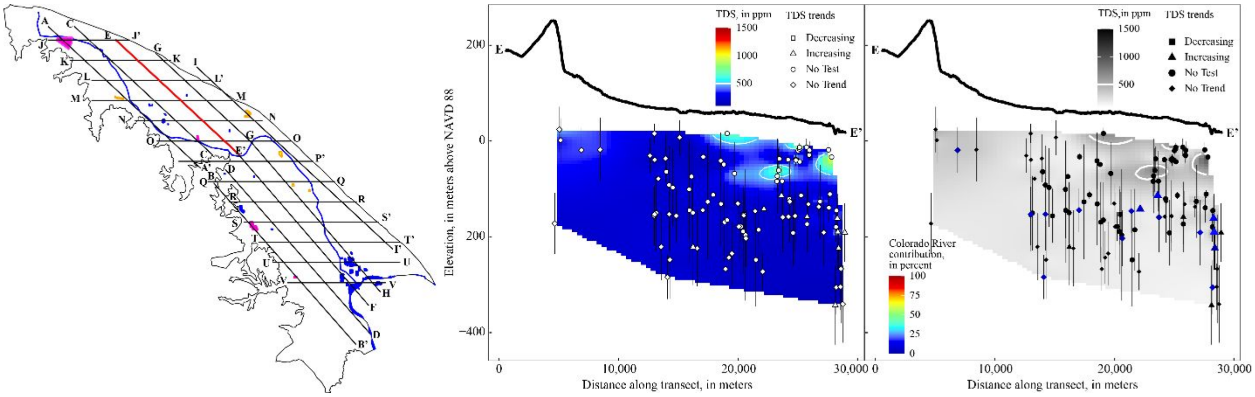
E
E'
TDS, in ppm
1500
1000
500
TDS trends
Decreasing
Increasing
No Test
No Trend
Colorado River contribution, in percent
100
75
50
25
0
Elevation, in meters above NAVD 88
Distance along transect, in meters
0
10,000
20,000
30,000

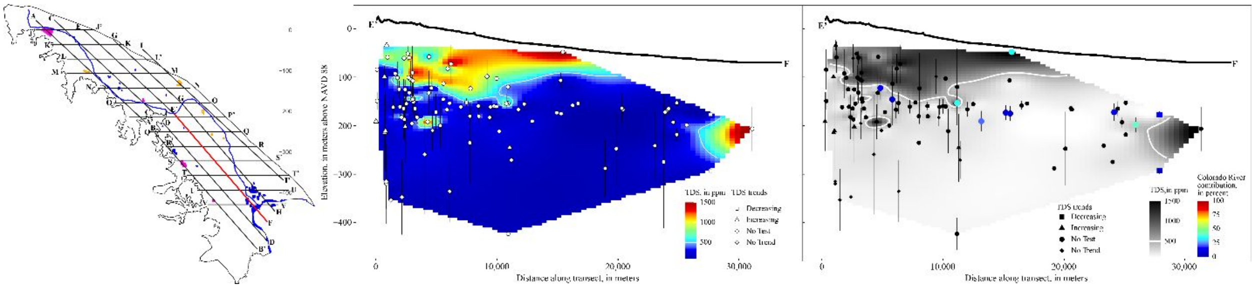
E'
F
Elevation, in meters above NAVD 88
Distance along transect, in meters
TDS, in ppm
TDS trends
Decreasing
Increasing
No Test
No Trend
Colorado River contribution, in percent
0
10,000
20,000
30,000

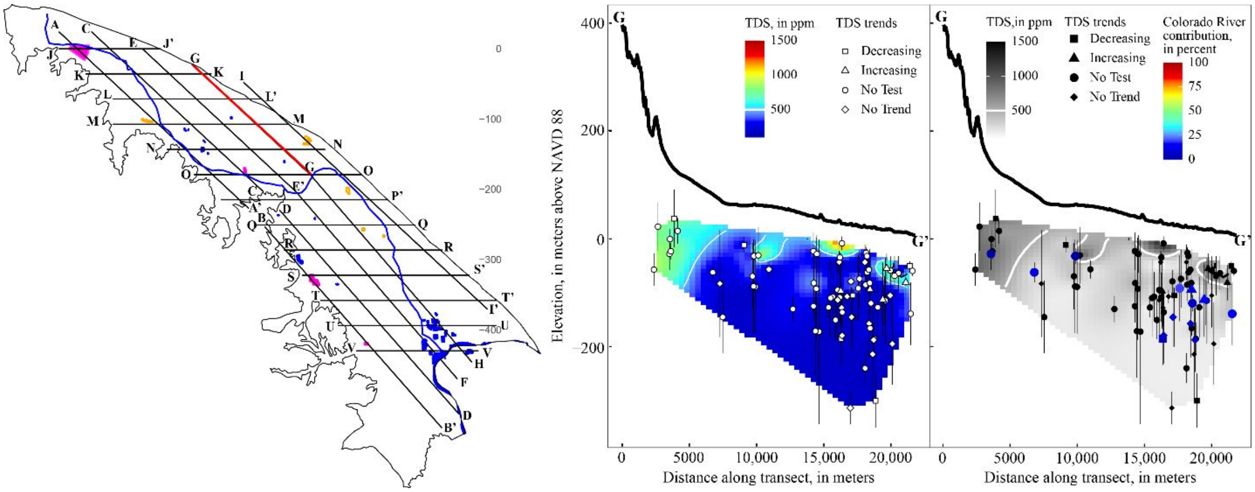
G
G'
TDS, in ppm
1500
1000
500
TDS trends
Decreasing
Increasing
No Test
No Trend
Colorado River contribution, in percent
100
75
50
25
0
Elevation, in meters above NAVD 88
400
200
0
−200
Distance along transect, in meters
0
5000
10,000
15,000
20,000

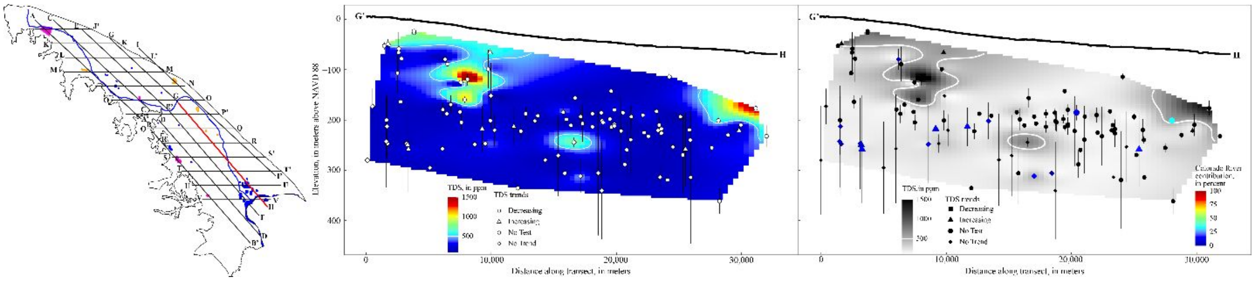
G'
H
Elevation, in meters above NAVD 88
Distance along transect, in meters
TDS, in ppm
TDS trends
Decreasing
Increasing
No Test
No Trend
Colorado River contribution, in percent
0
10,000
20,000
30,000

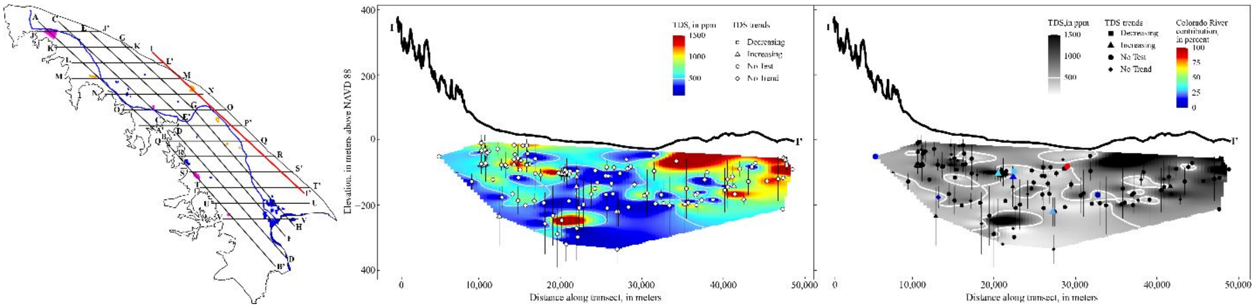
TDS, in ppm
TDS trends
Decreasing
Increasing
No Test
No Trend
Colorado River contribution, in percent
Elevation, in meters above NAVD 88
Distance along transect, in meters
I
I'

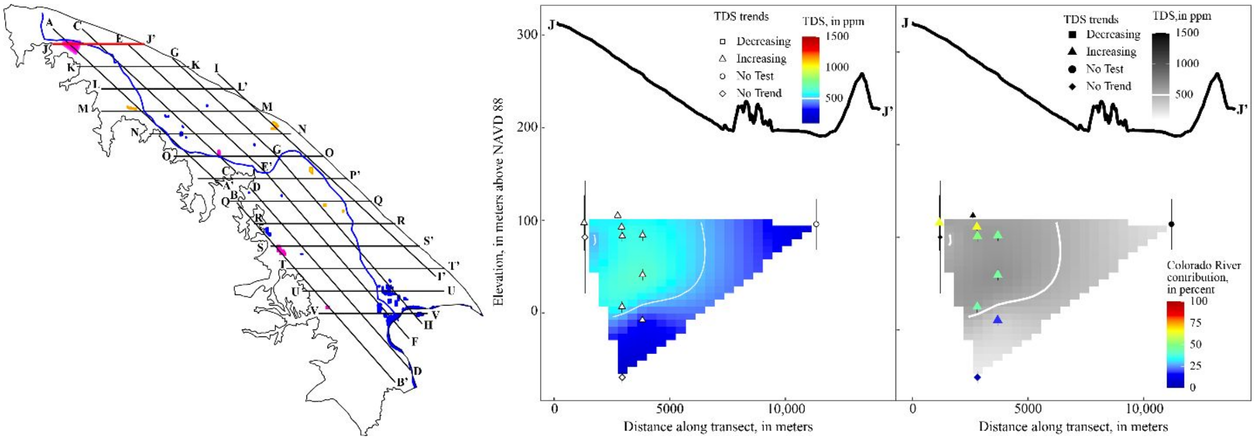
TDS trends
Decreasing
Increasing
No Test
No Trend
TDS, in ppm
Colorado River contribution, in percent
Elevation, in meters above NAVD 88
Distance along transect, in meters
J
J'

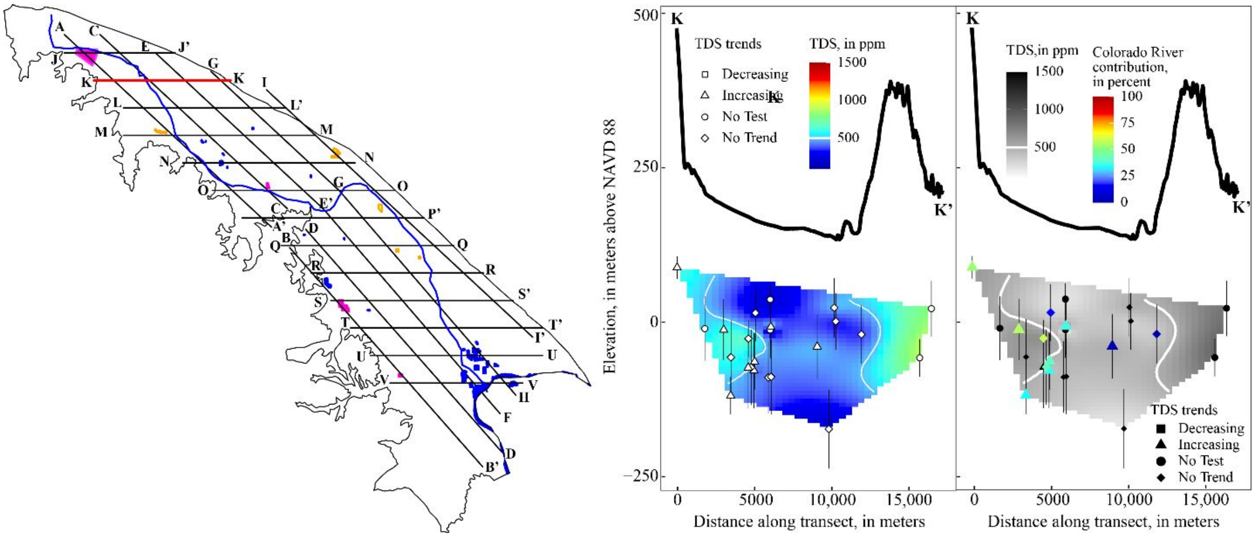
TDS trends
Decreasing
Increasing
No Test
No Trend
TDS, in ppm
Colorado River contribution, in percent
Elevation, in meters above NAVD 88
Distance along transect, in meters
K
K'

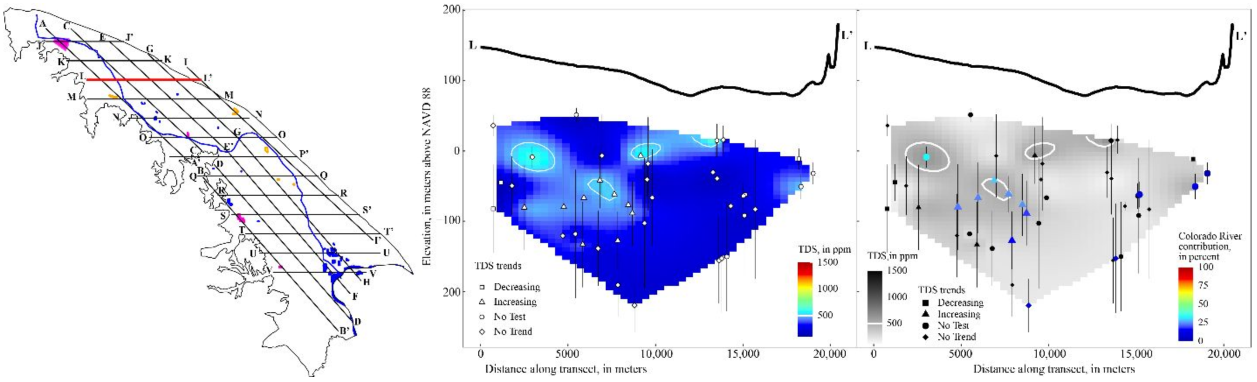
L
L'
Elevation, in meters above NAVD 88
Distance along transect, in meters
TDS trends
Decreasing
Increasing
No Test
No Trend
TDS, in ppm
Colorado River contribution, in percent

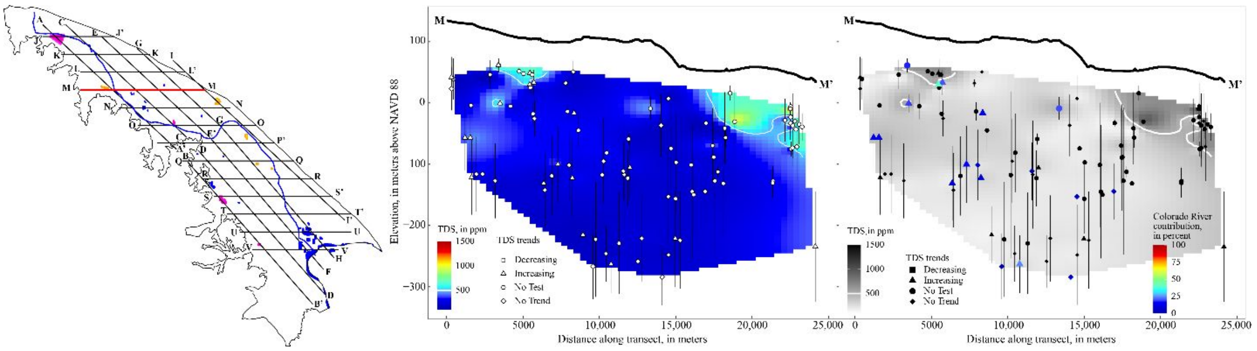
M
M'
Elevation, in meters above NAVD 88
Distance along transect, in meters
TDS, in ppm
TDS trends
Decreasing
Increasing
No Test
No Trend
Colorado River contribution, in percent

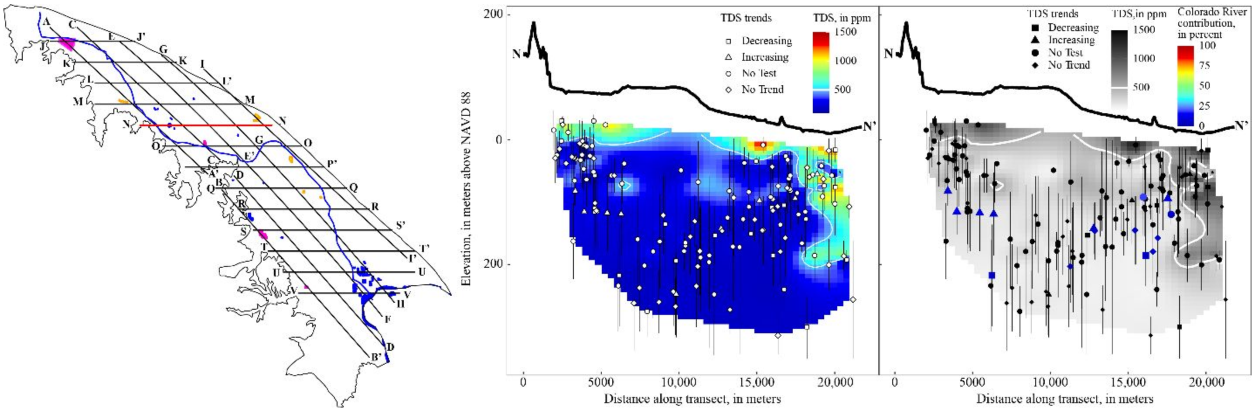
N
N'
Elevation, in meters above NAVD 88
Distance along transect, in meters
TDS trends
Decreasing
Increasing
No Test
No Trend
TDS, in ppm
Colorado River contribution, in percent

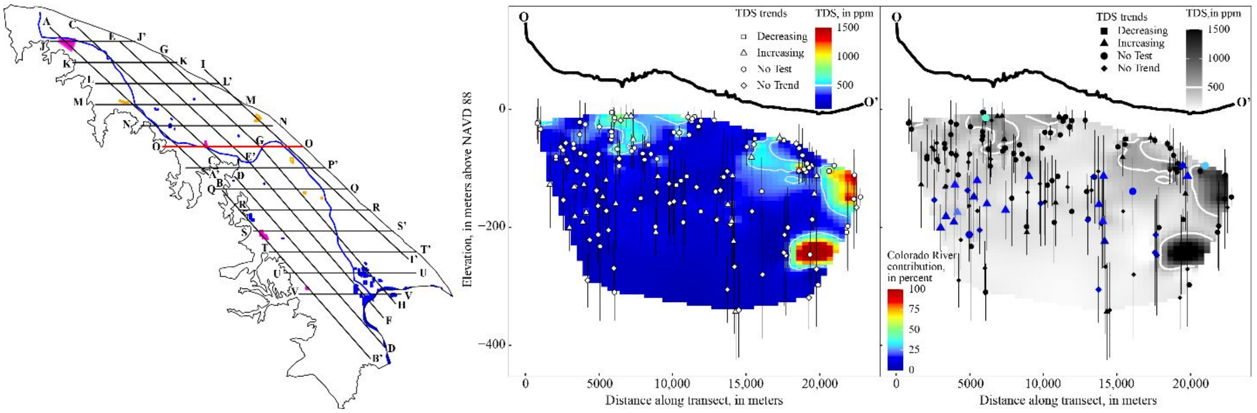
O
O'
TDS trends
Decreasing
Increasing
No Test
No Trend
TDS, in ppm
Colorado River contribution, in percent
Elevation, in meters above NAVD 88
Distance along transect, in meters

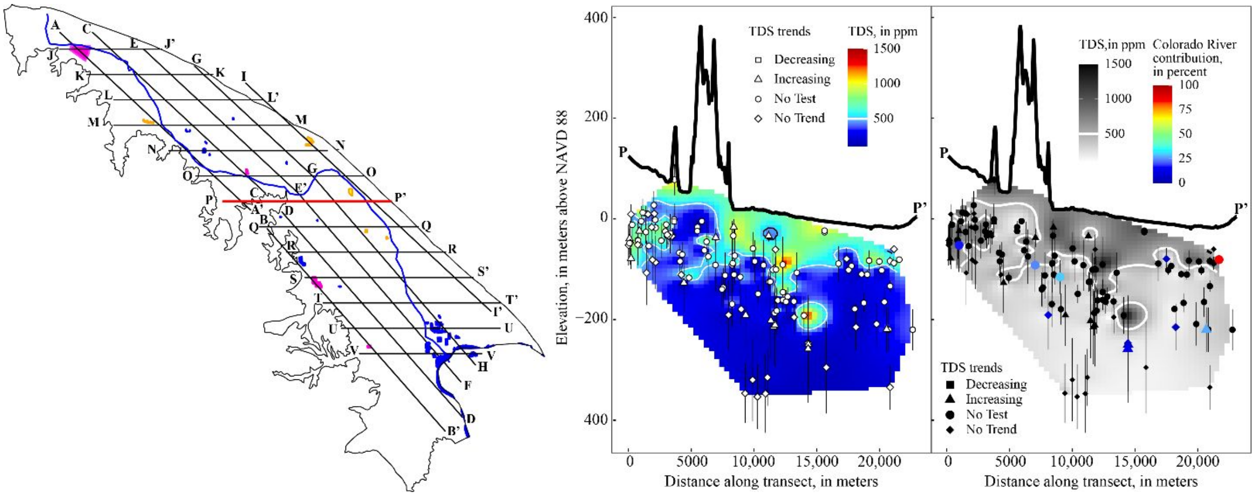
P
P'
TDS trends
Decreasing
Increasing
No Test
No Trend
TDS, in ppm
Colorado River contribution, in percent
Elevation, in meters above NAVD 88
Distance along transect, in meters

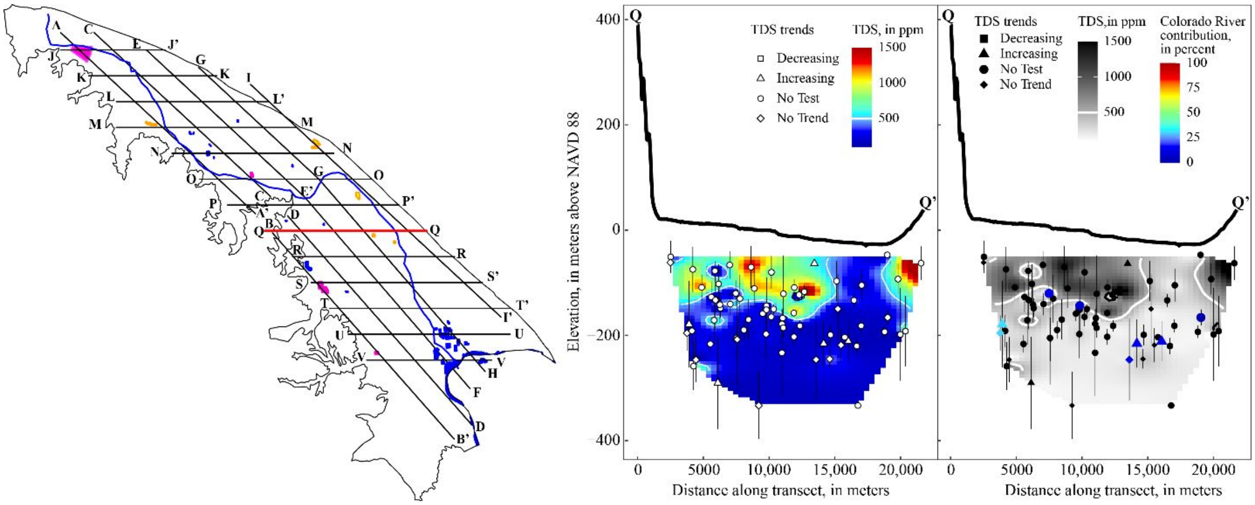
Q
Q'
TDS trends
Decreasing
Increasing
No Test
No Trend
TDS, in ppm
Colorado River contribution, in percent
Elevation, in meters above NAVD 88
Distance along transect, in meters

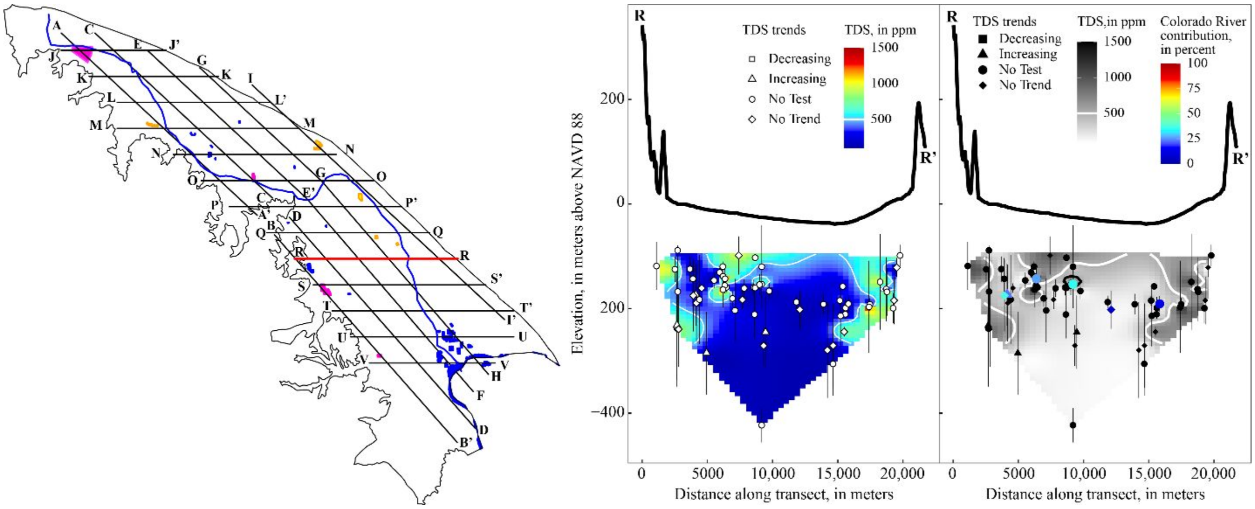
R
R'
TDS trends
Decreasing
Increasing
No Test
No Trend
TDS, in ppm
1500
1000
500
Colorado River contribution, in percent
100
75
50
25
0
Elevation, in meters above NAVD 88
Distance along transect, in meters

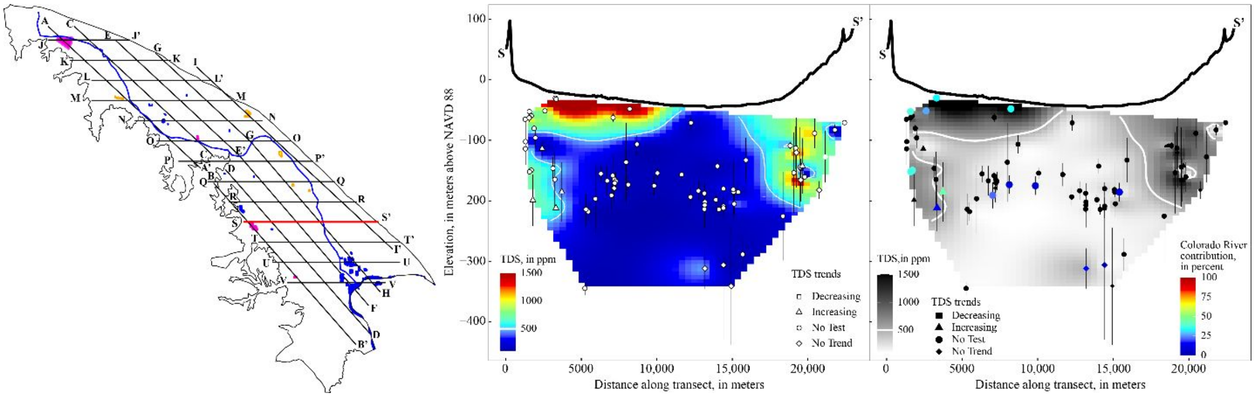
S
S'
TDS, in ppm
1500
1000
500
TDS trends
Decreasing
Increasing
No Test
No Trend
Colorado River contribution, in percent
Elevation, in meters above NAVD 88
Distance along transect, in meters

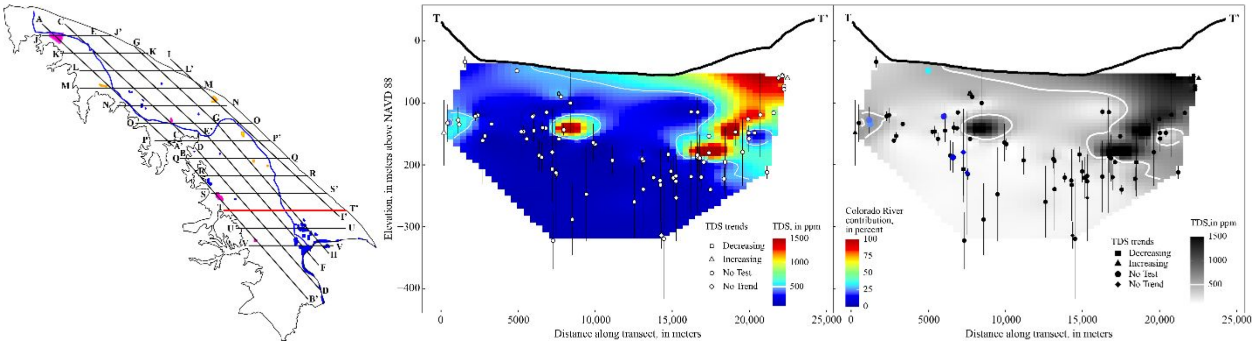
T
T'
TDS trends
Decreasing
Increasing
No Test
No Trend
TDS, in ppm
Colorado River contribution, in percent
Elevation, in meters above NAVD 88
Distance along transect, in meters

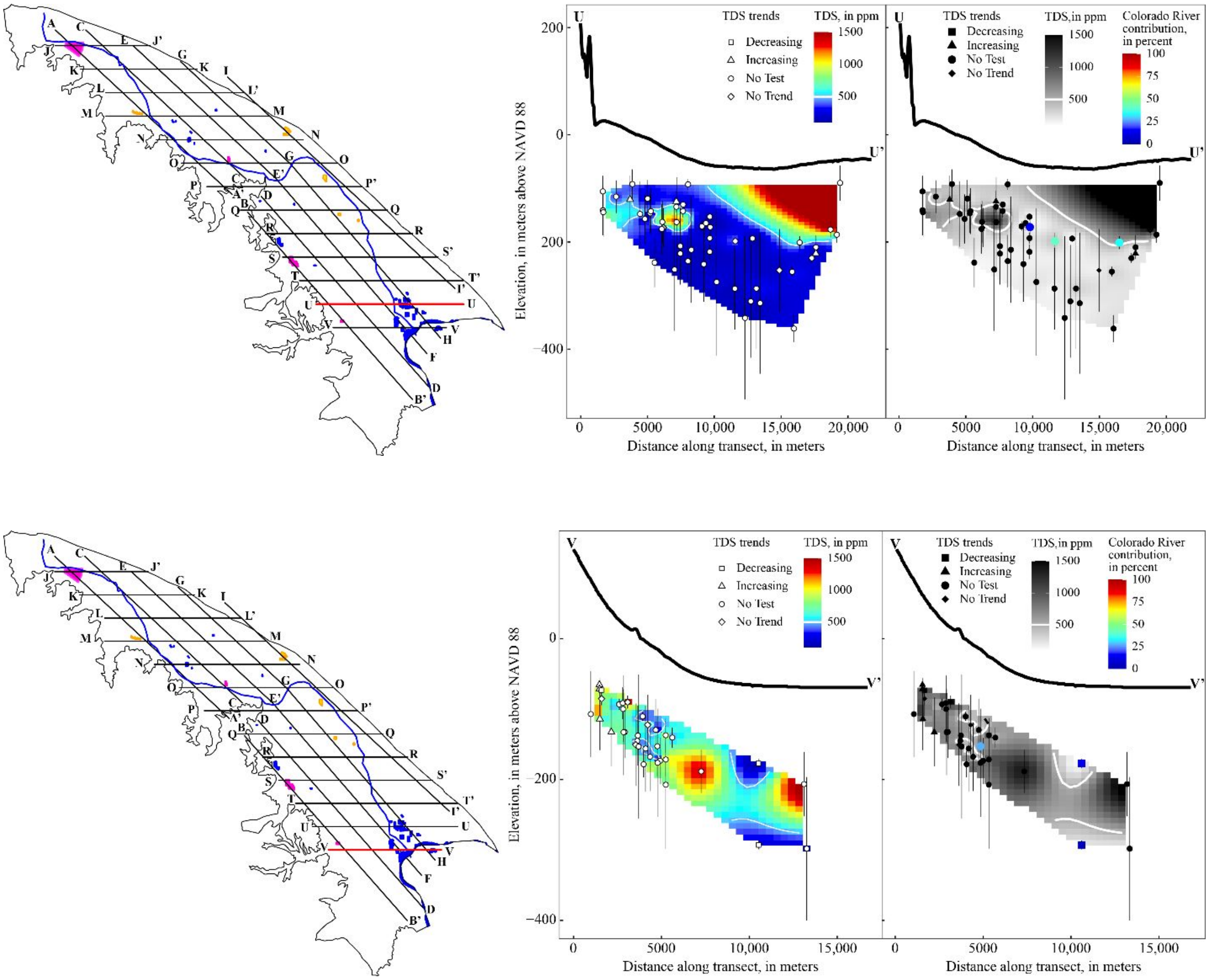

Figure S7. Two-dimensional cross-sections of portions of the aquifer system in the Indio subbasin, Coachella Valley, California, where there are wells with construction information showing interpolated TDS concentrations using the most recent measurement in a well, direction of TDS trends in the well, and modeled Colorado River water contribution for modeled wells. Location of each transect is shown in red in the insert map of the Indio subbasin.

3. References Cited in Supporting Information

1. Harkness, J.S., 2022, Groundwater database for a regional assessment of groundwater salinity variations and sources in the Indio Subbasin of the Coachella Valley, California: U.S. Geological Survey data release, https://doi.org/10.5066/P92DU67Q.
2. Rusydi, A.F., 2018, Correlation between conductivity and total dissolved solid in various type of water: A review. In *IOP conference series: earth and environmental science*, Vol. 118, p. 012019, http://doi.org/10.1088/1755-1315/118/1/012019.
3. Parkhurst, D.L., and Appelo, C.A.J., 2013, Description of input and examples for PHREEQC version 3—A computer program for speciation, batch-reaction, one-dimensional transport, and inverse geochemical calculations: U.S. Geological Survey Techniques and Methods, book 6, chap. A43, 497 p., http://pubs.usgs.gov/tm/06/a43/.

4. McCarthy, P.M., Jurgens, B.C., and Harkness, J.S., 2023, Inverse Model Data for: Salinity trends in a groundwater system supplemented by 50 years of managed aquifer recharge, https://doi.org/10.5066/P9KUBQKM.
5. Soldavini, A.L., Harkness, J.S., and Goldrath, D.A., 2022, Groundwater and surface water data for a regional assessment of groundwater salinity variations and sources in the Indio Subbasin of the Coachella Valley, California: U.S. Geological Survey data release, https://doi.org/10.5066/P9Z4ARSZ.
6. Soldavini, A. L., Goldrath, D., Shelton, J. L., Johnson, T., & Watson, E., 2021, Groundwater-quality data in the Coachella Valley Domestic Supply Aquifer Study Unit, 2020: Results from the California GAMA Priority Basin Project, U.S. Geological Survey Data Release, https://doi.org/10.5066/P9UYXI95.
7. U.S. Geological Survey, 2022, USGS water data for the Nation: U.S. Geological Survey National Water Information System database, https://doi.org/10.5066/F7P55KJN (accessed 31 March 2022).
8. California State Water Resources Control Board – Division of Drinking Water, 2022a, EDT Library and Water Quality Analyses Data and Download Page. https://www.waterboards.ca.gov/drinking_water/certlic/drinkingwater/EDTlibrary.shtml (accessed 31 March 2022).
9. California Department of Water Resources, 2022, Water Quality Data Download Page, https://data.cnra.ca.gov/dataset/water-quality-data (accessed 31 March 2022).
10. Coachella Valley Water District, Water Quality Data for Technical Memorandum No. 1 - Preliminary Data Review and Documentation of Technical Methods, 2015, http://web.cvwd.org/snmp/library/WQData-Share.xlsx (accessed 27 November 2020).
11. Robison, J.H., 1981, Data from geothermal wells near Oasis, lower Coachella Valley, California, U.S. Geological Survey Open-File Report 81-411, https://doi.org/10.2172/5045384.
12. Pebesma, E., 2018, Simple Features for R: Standardized Support for Spatial Vector Data. The R Journal 10 (1), 439-446, https://doi.org/10.32614/RJ-2018-009.
13. Gesch, D.B., Evans, G.A., Oimoen, M.J., Arundel, S., 2006, National Elevation Dataset (NED): U.S. Geological Survey database, accessed at https://www.usgs.gov/publications/national-elevation-dataset.
14. Finley, A., Banerjee, S., and Hjelle, Ø, 2017, MBA: Multilevel B-Spline Approximation. R package version 0.0-9. https://CRAN.R-project.org/package=MBA.
15. Sievert. Interactive Web-Based Data Visualization with R, plotly, and shiny. Chapman and Hall/CRC Florida, 2020.
16. U.S. Environmental Protection Agency Office of Ground Water and Drinking Water, 2020, Reductions of perchlorate in drinking water, EPA-815-F-20-002, https://www.epa.gov/sites/default/files/2020-05/documents/perchlorate_reductions_5.14.20.pdf (accessed 31 December, 2022).
